# Altered Lipid Profile Is a Risk Factor for the Poor Progression of COVID-19: From Two Retrospective Cohorts

**DOI:** 10.3389/fcimb.2021.712530

**Published:** 2021-09-30

**Authors:** Hui Jin, Junji He, Chuan Dong, Bin Li, Zhiyue Ma, Bilan Li, Tiande Huang, Jiangang Fan, Gang He, Xiaolong Zhao

**Affiliations:** ^1^ Department of Otolaryngology, Wuhan Third Hospital, Wuhan, China; ^2^ Department of Otolaryngology Head and Neck Surgery, Public Health Clinical Center of Chengdu, Chengdu, China; ^3^ Department of Otolaryngology Head and Neck Surgery, Sichuan Provincial People’s Hospital, University of Electronic Science and Technology of China, Chengdu, China

**Keywords:** COVID-19, dyslipidemia, recurrence, lipid-lowering therapy, prognosis

## Abstract

**Background:**

The coronavirus disease 2019 (COVID-19) pandemic has spread worldwide. However, the impact of baseline lipid profile on clinical endpoints in COVID-19 and the potential effect of COVID-19 on lipid profile remain unclear.

**Methods:**

In this retrospective cohort study, we consecutively enrolled 430 adult COVID-19 patients from two Chinese hospitals (one each in Chengdu and Wuhan). The lipid profile before admission and during the disease course and the clinical endpoint including in-hospital death or oropharyngeal swab test positive again (OSTPA) after discharge were collected. We used Kaplan–Meier and Cox regression to explore the lipid risk factors before admission associated with endpoints. Then, we assessed the lipid level change along with the disease course to determine the relationship between pathology alteration and the lipid change.

**Results:**

In the Chengdu cohort, multivariable Cox regression showed that low-density lipoprotein cholesterol (LDL-C) dyslipidemia before admission was associated with OSTPA after discharge for COVID-19 patients (RR: 2.51, 95% CI: 1.19, 5.29, p = 0.006). In the Wuhan cohort, the patients with triglyceride (TG) dyslipidemia had an increased risk of in-hospital death (RR: 1.92, 95% CI: 1.08, 3.60, p = 0.016). In addition, in both cohorts, the lipid levels gradually decreased in the in-hospital death or OSTPA subgroups since admission. On admission, we also noticed the relationship between the biomarkers of inflammation and the organ function measures and this lipid level in both cohorts. For example, after adjusting for age, sex, comorbidities, smoking, and drinking status, the C-reactive protein level was negatively associated with the TC lipid level [β (SE) = -0.646 (0.219), *p* = 0.005]. However, an increased level of alanine aminotransferase, which indicates impaired hepatic function, was positively associated with total cholesterol (TC) lipid levels in the Chengdu cohort [β (SE) = 0.633 (0.229), *p* = 0.007].

**Conclusions:**

The baseline dyslipidemia should be considered as a risk factor for poor prognosis of COVID-19. However, lipid levels may be altered during the COVID-19 course, since lipidology may be distinctly affected by both inflammation and organic damage for SARS-CoV-2.

## Introduction

In December 2019, a new viral disease was identified in China, termed coronavirus disease 2019 (COVID-19), which has since spread rapidly throughout the world until now (Huang et al., 2020). A novel beta-coronavirus, known as severe acute respiratory syndrome corona virus 2 (SARS-CoV-2), was identified as the COVID-19 pathogen and is known to trigger severe pneumonia and acute, often lethal, lung failure (Huang et al., 2020). So far, 130 million confirmed cases of COVID-19 and about 3 million deaths had been reported (World Health Organization, 2021). In addition, many discharged COVID-19 patients had positive oropharyngeal swab tests for SARS-CoV-2 RNA (Chen et al., 2020). COVID-19 oropharyngeal swab test positive again (OSTPA) after discharge or high mortality will inevitably impose a significant burden on healthcare systems due to not only treatment but also the increased need for supervision after discharge (Hoang et al., 2020). Therefore, it is important to identify risk factors for the poor progression of COVID-19. It is also essential that COVID-19 patients are advised regarding how to reduce the risk of recurrence such as the lifestyle change.

Multiple cardiovascular metabolic abnormalities included diabetes, obesity, hypertension, and dyslipidemia (Lakka et al., 2002). These are all important risk factors for cardiovascular disease, which is one of the leading causes of morbidity worldwide and is anticipated to rise substantially over the next decades (Zhao et al., 2019a; Zhao et al., 2019b). On admission, 20%–51% of COVID-19 patients reported at least one metabolic comorbidity, with the most common being diabetes (10–20%) and hypertension (10–15%) (Cai et al., 2020; Chen et al., 2020; Guan et al., 2020; Guo et al., 2020; Gao et al., 2020; Mehra et al., 2020). A retrospective multicenter study conducted in China revealed that patients with these comorbidities (based on self-report) on admission experienced poorer clinical endpoints, which consisted of admission to the intensive care unit (ICU), or invasive ventilation, or death, than those without comorbidities (Guan et al., 2020). However, two studies from Wuhan indicated that serum lipid levels were unexpectedly lower in COVID-19 patients compared to healthy subjects (Fan et al., 2020; Wei et al., 2020). The reason may be that the COVID-19 pathology might potentially have substantial influence on lipidology in the patients (Radenkovic et al., 2020). The prominent pathological changes in COVID-19 include systematic inflammation; multiple-organ injury in the cardiovascular system, the liver, and the kidney; and other life-threatening pathologies, and then may result in the lipidology change in COVID-19 patients (Chen et al., 2020). In this context, it was recently even suggested that lipidology-COVID-19 bidirectional interactions should also be considered, which may help to guide clinical decisions, with the recommendation for patients at higher cardiovascular risk but with normal lipid levels to continue lipid-lowering therapy unless absolutely contraindicated, particularly in primary prevention settings.

## Methods

### Study Design and Subjects

**Figure 1:**
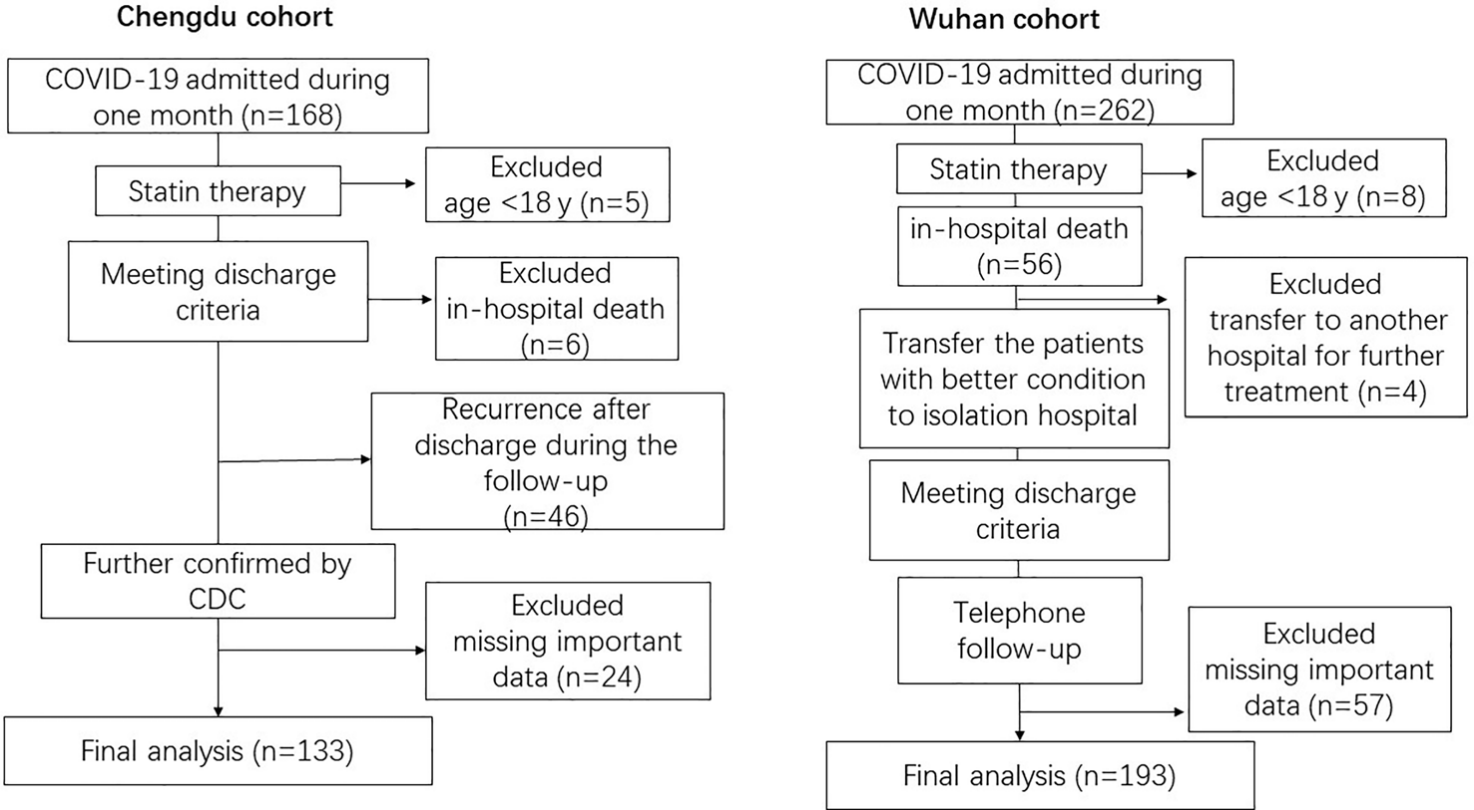
Study flow diagram. Centers for Disease Control (CDC).

This retrospective cohort study included two cohorts of adult subjects (aged ≥18 years) recruited from the Public Health Clinical Center of Chengdu (Chengdu cohort) and the Wuhan Third Hospital (Wuhan cohort). All adult patients diagnosed with COVID-19 according to the World Health Organization (WHO) interim guidelines were screened in February and then monitored until April 30, 2020. A total of 430 adult patients were hospitalized from the two hospitals in February. The final analysis included 326 patients with complete data for all clinical variables (see **Figure 1**). The study was conducted in accordance with the Declaration of Helsinki and received approval from the local internal review board of the institutional ethics committee.

### Definitions

The primary endpoint was here defined as the recurrence after discharge as positive oropharyngeal swab tests for SARS-CoV-2 RNA after discharge or in-hospital death according to the Chinese management guideline for COVID-19 (version 7.0) (Wei, 2020). The secondary endpoint was admission to the ICU or the need for invasive ventilation. These endpoints were used as they represent the most serious outcomes of COVID-19 infection and have been employed in previous H7N9 reports (Gao et al., 2013; Guan et al., 2020; Zeng et al., 2020). The indications for discharge included the absence of fever for at least 3 days, substantial improvement in both lungs based on chest CT, clinical remission of respiratory symptoms, and two throat swab samples negative for SARS-CoV-2 RNA (interval of at least 24 h) (Wei, 2020). The recurrence after discharge was confirmed by the Centers for Disease Control (CDC) in China, if the routinely daily RT-PCR test was positive or/and any respiratory symptoms appeared. In order to reduce the false negative or positive rate of nucleic acid tests, running multiple tests and collecting different combined specimens from both oropharyngeal and nasal swabs would be effective approaches to diminish the possibility. Critical care pharmacists or doctors, however, routinely contacted the patients and families to elicit information regarding chronic medications, including prior lipid-lowering therapy prescriptions. During the hospitalization period, critical care pharmacists or doctors continued lipid-lowering therapy when previously prescribed or started a new statin prescription for recognized indications (for example, newly diagnosed dyslipidemia, an acute coronary syndrome, myocardial infarction, or stroke) (Stone et al., 2013 2014). Regarding lipid-lowering therapy status, patients were classified as never previously prescribed lipid-lowering therapy (without therapy previously group) or current lipid-lowering therapy and newly prescribed during the hospitalization (with therapy group). Intensity of statin therapy was classified into high-intensity, moderate-intensity, and low-intensity, according to the 2013 ACC/AHA guidelines (Stone et al., 2014).

### Anthropometric and Biochemical Measurements

The body mass index (BMI) was calculated as weight in kilograms divided by height in meters squared (kg/m^2^). Daytime blood pressure (BP) was measured as suggested in the American Society of Hypertension Guidelines (Zhao et al., 2018). The patients had a routine laboratory lipid test before infection and in the hospital during the following time points: (1) before February 1, 2020, as the annual regular medical examination [14 (10,17) days before the time on admission due to their SARS-CoV2 infection as pre-infection (PI)] and (2) on admission (OA) (between February 1, 2020, and then monitored until April 30) (set as day 0) after being tested SARS-CoV-2 positive during the COVID-19 epidemic in Wuhan. In addition, all patients had the lipid data available during the disease progression or treatment [8 (3, 9) days after the time on admission as the time in hospitalization (IH)]. All patients had the lipid data available 1–3 days before the time of discharge when they recovered or in-hospital death [15 (9, 23) days after the time on admission] as the endpoint time. For each subject, serum lipid profiles were obtained, including total cholesterol (TC), triglycerides (TG), high-density lipoprotein cholesterol (HDL-C), and low-density lipoprotein cholesterol (LDL-C). The criteria for dyslipidemia or hyperlipidemia were TC ≥ 5.17 mmol/l or LDL-C ≥ 3.33 mmol/l, TG ≥ 150  mg/dl (1.7  mmol/l), or HDL-C < 40 mg/dl (1.0 mmol/l) in men/<50  mg/dl (1.3  mmol/l) in women in accordance with the criteria of the United States National Cholesterol Education Program Adult Treatment Panel III (NCEPIII) (Grundy et al., 2002). Routine blood tests were performed to assess the blood count profile and to determine the levels of aspartate aminotransferase (AST), alanine aminotransferase (ALT), and creatinine, as an indicator of renal and liver function. The other serum markers measured included creatine kinase, C-reactive protein (CRP), lactate dehydrogenase, myocardial enzymes, and procalcitonin. All patients were assessed by chest computed tomography (CT).

### Statistical Analysis

Normal, skewed, and categorical data are presented as means and standard deviations, medians with interquartile range, and percentages, respectively. Differences in baseline characteristics among unrelated subgroups were examined using the Kruskal–Wallis H-test or Mann–Whitney U test, one-way analysis of variance, Fisher’s exact test, or χ^2^ test according to the data distribution. The paired non-parametric test is used to compare the difference among different stages in the same cohort subgroup of patients. *p*-values for trends were calculated using the linear-by-linear association test for dichotomous variables.

Each cohort data were used separately for analysis. Kaplan–Meier analysis was used to estimate survival endpoints. Univariable and multivariable Cox proportional hazard regression models were then used to determine the potential risk factors for the primary endpoints. Risk ratios (RRs) and 95% confidence intervals (CIs) were also reported. Cox regression was considered more appropriate than logistic regression because the former can account for the potential impact of variation in follow-up times among patients; multivariable logistic regression was used to explore the risk factors for the second endpoints. The data are presented as odds ratio (OR) (95% CI). Stepwise multivariate linear regression was used to identify laboratory biomarkers associated with the lipid profile on admission. The proportionality assumption was checked with plots of scaled Schoenfeld residuals against transformed time and a goodness-of-fit test using the sample size. Proportionality was not violated. All analyses were performed using SPSS software (version 20.0; SPSS Inc., Chicago, IL, USA). In all analyses, *p* < 0.05 was considered statistically significant.

### Patient and Public Involvement

Patients or the public WERE NOT involved in the design, or conduct, or reporting, or dissemination plans of our research.

## Results

### Baseline Characteristics for COVID-19 Patients

The study included 326 adult COVID-19 patients positive for SARS-CoV-2 RNA (133 patients from the Chengdu cohort and 193 from the Wuhan cohort). The study flow diagram is shown in **Figure 1**. Compared to the Chengdu cohort patients, those in the Wuhan cohort were older and more commonly presented with respiratory or systematic symptoms (e.g., fever, cough, sputum production, shortness of breath, fatigue, or diarrhea) (**Table 1**). In all patients, the lipid level difference between COVID-19 patients with comorbidities and those without is shown in **Supplementary Table S1**, and we found that lipid levels (TC, TG, LDL-c) on admission were higher in the patients with metabolic diseases (obesity, hypertension, diabetes) compared with the patients without these diseases.

**Table 1:** Demographic and clinical characteristics in the two cohorts.

	All patients	Chengdu cohort	Wuhan cohort	p-value
	n = 326	n = 133	n = 193	
Age, years	52.5 ± 0.9	44.9 ± 1.4	58.1 ± 1.0	p < 0.001
BMI, kg/m^2^	23.7 ± 0.2	23.3 ± 0.3	24.0 ± 0.2	0.132
No. of males, %	52.6	51.4	53.3	0.731
Smoking status, %	16.7	15.5	15.6	0.633
Drinking status, %	19.9	23.9	17.8	0.231
**Symptoms, %**				
Fever on admission	37.2 ± 0.1	36.9 ± 0.1	37.4 ± 0.1	p < 0.001
Temperature on admission				
>37.3	45	23.2	60.4	p < 0.001
Highest temperature (°C)	38.1 ± 0.1	37.6 ± 0.1	38.2 ± 0.1	p < 0.001
Nasal congestion	0.5	0.8	0.6	0.866
Headache	6.7	7.7	6.1	0.602
Cough	77.5	60.6	89.3	p < 0.001
Sore throat	11.1	13.4	9.6	0.347
Sputum production	43	34.5	48.2	0.009
Fatigue	41.5	23.2	53.8	p < 0.001
Shortness of breath	38.3	12	56.3	p < 0.001
Nausea or vomiting	1.8	0.7	2.5	0.194
Diarrhea	12	3.5	18.3	p < 0.001
Chill	8.5	8.5	8.6	0.886
**Comorbidity, %**				
Renal disease	1.8	1.5	2	0.400
Pulmonary disease	1.2	1.1	1.5	0.555
Heart disease	5.7	5.4	5.9	0.908
Hepatic disease	9.7	9.9	10.0	0.365
Intracranial disease	2.3	2.8	2	0.638
Hypertension	28.1	26.3	27.5	0.847
Obesity	8.2	7.5	8.6	0.432
Diabetes	9.1	8.8	9.6	0.698
Other diseases	0.6	0.5	0.7	0.816
**Lipid profiles on admission**				
TC, mmol/l	3.83 (3.21,4.55)	4.2 (3.41,5)	3.65 (3.13,4.26)	p < 0.001
TG, mmol/l	1.19 (0.90,1.71)	1.34 (0.88,2.03)	1.12 (0.90,1.53)	p < 0.001
LDL-C, mmol/l	2.82 (2.39,3.34)	2.78 (2.31,3.27)	2.86 (2.45,3.45)	0.322
HDL-C, mmol/l	1.01 (0.82,1.29)	1.29 (1.07,1.52) 6	0.88 (0.75,1.02)	p < 0.001
**Disease severity, %**				p < 0.001
Mild cases	6.4	11.1	0	
Moderate cases	59.1	63	59.4	
Severe cases	11.1	11.9	11.2	
Critical cases	23.4	14.1	29.4	
**The laboratory findings**				
White blood cell count, 10^9^/l				0.001
<4	18.1	9.2	24.9	
4–10	73.1	81.7	67	
>10	8.8	9.2	8.1	
Neutrophil count, 10^9^/l				0.015
<2	12.9	9.2	15.7	
2–7	74.3	82.4	68.5	
>7	12.9	8.5	15.7	
C-reactive protein, >5 mg/l	70.8	48.6	86.3	p < 0.001
Lymphocyte count, 10^9^/l				p < 0.001
<0.8	50	22.5	69	
0.8–4	49.7	76.8	31	
>4	0.3	0.7	0	
Platelet count, 10^9^/l				p < 0.001
<100	16.1	5.6	22.8	
100–300	78.1	86.6	73.1	
>300	5.8	7.7	4.1	
D-dimer, >0.5 mg/l	40.1	18.4	54.8	p < 0.001
Alanine aminotransferase, >40 U/l	21.3	24.6	19.3	0.236
Aspartate aminotransferase, >40 U/l	21.6	14.8	26.4	0.010
Creatinine, μmol/l				0.009
<40	2.9	3.5	2.5	
40–133	88	93	84.3	
>133	9.1	3.5	13.2	
Lactate dehydrogenase, U/l	277.16 ± 10.08	218.29 ± 7.68	319.55 ± 15.78	p < 0.001
<109	1.2	1.4	1	
109–245	57.3	76.8	44.2	
>109	41.5	21.8	54.8	
High-sensitive cardiac troponin I, ng/ml	6.24 ± 1.72	17.14 ± 4.59	0.08 ± 0.03	p < 0.001
Procalcitonin, >0.5 μg/l	25.6	2.8	40.6	p < 0.001
Fasting glucose, mmol/l	6.19 ± 0.13	5.55 ± 0.09	6.66 ± 0.21	p < 0.001
**Imaging features, %**				p < 0.001
Normal	0.6	1.4	0	
Local patchy shadowing	13.5	24.6	5.6	
Bilateral patchy shadowing	85.9	71.8	94.4	
**Treatments, %**				
Antibiotics	64.9	23.2	94.4	p < 0.001
Antiviral treatment	98.0	99.3	97	0.135
Corticosteroids	17.7	8.9	65.2	p < 0.001
Intravenous immunoglobulin	25.1	1.4	41.4	p < 0.001
Antifungal medications	1.8	2.1	1	0.408
Oxygen therapy	87.7	98.6	79.7	p < 0.001
Mechanical ventilation	21.3	11.3	27.4	p < 0.001
Extracorporeal membrane oxygenation	1.4	1.4	0	NA
ICU admission	28.9	42.3	18.3	p < 0.001
**Lipid-lowering therapy before admission**, %	22.1	18.8	24.4	0.235
**Lipid-lowering therapy during admission**				
Statin therapy, %	15.33	14	16	0.421
High-intensity statin therapy, %		4	4.7	
Moderate-intensity statin therapy, %		6	7.3	
Low-intensity statin therapy, %		4	4	
**Disease period**				
Time from symptom onset to admission, days	7.8 ± 5.3	6.2 ± 6.5	9 ± 5.4	p < 0.001
The first hospital length of stay, days	17.4 ± 10.5	20.5 ± 12.0	15.2 ± 7.5	p < 0.001
The second hospital length of stay, days		14.2 ± 6.6	NA	NA
The recurrence time after discharge, days		18.2 ± 7.1	NA	NA
Time from symptom onset to the endpoint, days	29.6 ± 16.9	37.1 ± 21.8	24.2 ± 8.6	p < 0.001
**Complications, %**				
Septic shock	3.6	0.7	5.6	0.020
Acute respiratory distress syndrome (ARDS)	10.08	11.1	10.8	0.915
Acute kidney injury	1.2	0.7	1.5	0.491
Disseminated intravascular coagulation	0.3	0.7	0	0.238
Pneumonia	80.5	69.7	92.9	p < 0.001
Multiple organ failure	0.9	0	1.5	0.341
**Clinical endpoints, %**				0.001
Deaths in hospital			28.9	
Discharge from hospital		67.6	71.1	
Recurrence rate		32.4		

The data with normal distribution are presented as means (SD); the skewed data are presented as the median (IQR), and categorical data as the number (percentage). Differences in baseline characteristics among the subgroups were examined using the Mann-Whitney U test was used to compare differences between groups or the χ^2^ test according to the characteristics of the data distribution. NA, not available.

BMI, body mass index; ICU, intensive care unit; TC, total cholesterol; TG, triglycerides; HDL-C, high-density lipoprotein cholesterol; LDL-C, low-density lipoprotein cholesterol.

The Wuhan cohort patients tended to have more severe COVID-19 and abnormal laboratory and chest CT results (**Table 1**). Interestingly, the Wuhan patients had lower lipid levels (TC, TG, and HDL-C) on admission (**Table 1**). However, we observed that there were no relationships between dyslipidemia on admission and COVID-19 severity in either cohort (**Supplementary Tables S2, S3**). There was a linear trend between COVID-19 severity and the number of laboratory biomarkers of lymphopenia, inflammation [i.e., CRP, procalcitonin], and organ function measures [i.e., AST, ALT or creatinine) on admission in both cohorts (**Supplementary Tables S2, S3**).

Before admission, some patients had received lipid-lowering therapy but data about the specific indications for the prescription and the dose were not available and are unknown. After admission, critical care pharmacists or doctors continued lipid-lowering therapy or started a new statin prescription for recognized indications with differently prescribed doses. The rates of lipid-lowering therapy before and after admission in both cohorts are shown in **Table 1**.

### The Relationship Between the Pathology Alteration due to COVID-19 and Lipid Profile on Admission

Inflammation and impaired organ function are two critical pathology aspects of COVID-19. To observe the effect of COVID-19 on the blood lipid levels, we used a multivariate linear regression model to determine factors independently associated with the serum lipid profile in the same time point. Interestingly, the correlations of inflammatory biomarkers (CRP and procalcitonin) with lipid levels were different from those for biomarkers of organ function (AST, ALT, and creatinine). For example, after adjusting for age, sex, comorbidities, smoking, and drinking status, the CRP level was negatively associated with the TC lipid level [β (SE) = -0.646 (0.219), *p* = 0.005]. However, an increased level of ALT, which indicates impaired hepatic function, was positively associated with TC lipid levels in the Chengdu cohort [β (SE) = 0.633 (0.229), *p* = 0.007] (**Table 2**). Similar correlations were seen for other lipids in the both cohorts, including TC, TG, HDL-C, and LDL-C. In summary, it is important to note that we observed that the inflammatory biomarkers were negatively associated with lipid level but the biomarkers of hepatic and kidney organ dysfunction were positively associated with lipid level (**Table 2**).

**Table 2:** The relationship between lipid level and biomarkers of pathological changes in COVID-19 for two cohorts, respectively.

	TC	p value	TG	p value	HDL	p value	LDL	p value
**Chengdu cohort**								
C-reactive protein, mg/l	-0.646 (0.219)	0.005			-0.224 (0.049)	0.001		
Procalcitonin, μg/l								
Alanine aminotransferase, U/l	0.633 (0.229)	0.007					0.524 (0.144)	0.001
Aspartate aminotransferase, U/l					0.173 (0.075)	0.023		
Creatinine, μmol/l								
Lactate dehydrogenase, U/l								
High-sensitive cardiac troponin I, ng/ml								
**Wuhan cohort**								
C-reactive protein, mg/l								
Procalcitonin, μg/l	-0.338 (0.135)	0.013			-0.163 (0.040)	0.001	-0.251 (0.096)	0.010
Alanine aminotransferase, U/l			0.169 (0.085)	0.048				
Aspartate aminotransferase, U/l								
Creatinine, μmol/l			0.244 (0.056)	0.001				
Lactate dehydrogenase, U/l								
High-sensitive cardiac troponin I, ng/ml								

The data are presented as β (SE). The multivariate linear regression model was adjusted for age, BMI, males (*vs.* female), smoking and drinking status (*vs.* not present), and various kinds of comorbidities (e.g., hypertension, chronic renal disease, pulmonary disease, heart disease, hepatic disease) and the use of lipid-lowering therapy before admission.

TC, total cholesterol; TG, triglycerides; HDL-C, high-density lipoprotein cholesterol; LDL-C, low-density lipoprotein cholesterol.

### The Treatments and the Clinical Endpoints

During hospitalization, the complication rates of septic shock and pneumonia in the Wuhan cohort were significantly higher compared to those in the Chengdu cohort (5.6% *vs.* 0.7% and 92.9% *vs.* 69.7%, respectively). In the Wuhan cohort, more patients received treatment with antibiotics, corticosteroid intravenous immunoglobulin, and mechanical ventilation compared to those in the Chengdu cohort (94.4% *vs.* 23.2%; 65.2% *vs.* 8.9%; 41.4% *vs.* 1.4%; and 27.4% *vs.* 11.3%, respectively). However, the patients in the Chengdu cohort had greater access to ICUs and oxygen therapy (**Table 1**).

In the Chengdu cohort, the mean length of stay (in the first hospital) was 20.5 days. The longest recorded stay was 75 days. Because the sample size of in-hospital death was too small for statistical analysis, these patients were excluded (n = 6), and we focused on the recurrence of COVID-19 after discharge. Oropharyngeal swab samples showed that 32.4% of patients were positive for SARS-CoV-2 RNA after discharge. The mean recurrence time from the first discharge in the Chengdu cohort was 18 days. Following readmission, the mean length of stay was 14 days. Overall, the mean time from the onset of illness (before admission) to first discharge in the Chengdu patients was 37 days (**Table 1**).

In the Wuhan cohort, 28.9% of patients died during hospitalization and 137 survived patients were transferred to isolation hospitals for at least 14 days for further monitoring in accordance with the health policy and then discharged. The longest delay to first admission was 60 days in the Wuhan cohort. The mean time from initial symptom onset to death was 24 days (**Table 1**). Telephone follow-up interviews showed that no other discharged patients had died in the Wuhan cohort.

### The Relationship Between Lipid Profile Before Admission and the Clinical Endpoints

**Figure 2:**
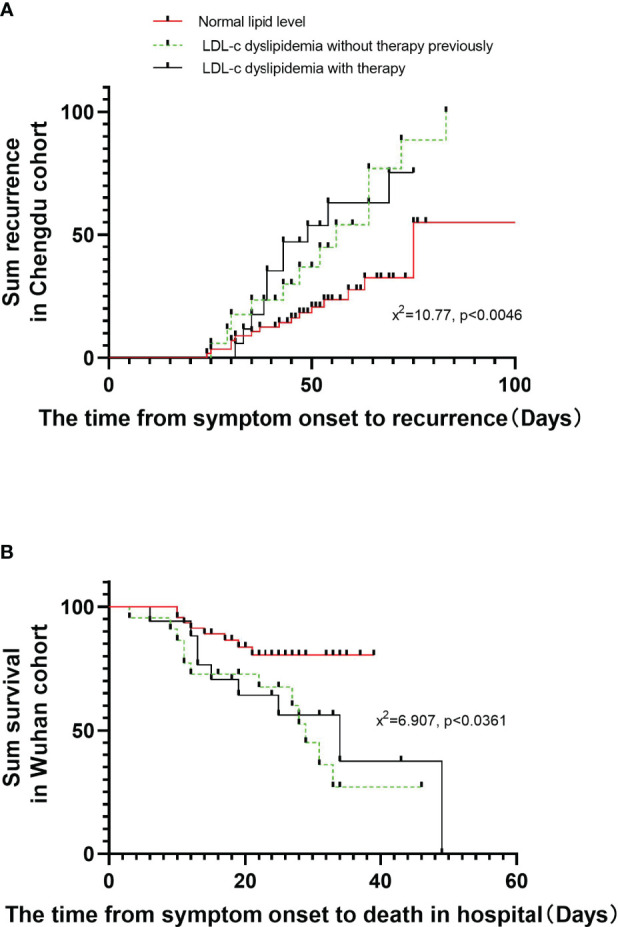
Comparison of the time-dependent risk of reaching to the recurrence after discharge or in-hospital death. The time-dependent risk of reaching to the recurrence after discharge in COVID-19 patients with dyslipidemia or lipid-lowering therapy compared with the control in the Chengdu cohort **(A)**. **(B)** shows the time-dependent risk of reaching to the in-hospital death in COVID-19 patients with dyslipidemia or lipid-lowering therapy compared with the control in the Wuhan cohort. Kaplan–Meier analysis was used to estimate and plot survival endpoints among subgroups, with the log-rank test being reported.

**Figure 2** shows Kaplan–Meier estimates of the risk of the primary endpoints. The risk factors assessed included TC, TG, HDL, and LDL-C dyslipidemia present before admission. We chose the patients without any dyslipidemia or the lipid-lowering therapy previously as the control and found patients with LDL-C dyslipidemia with or without lipid-lowering therapy had a significantly higher risk of COVID-19 recurrence after discharge compared to the control patients, as revealed by a log-rank test (X^2^ = 10.77, *p* = 0.0046) (**Figure 2A**). Previous studies reported that laboratory results on admission (specifically ALT, lactate dehydrogenase, high-sensitivity cardiac troponin I, creatine kinase, and d-dimer levels) may be risk factors for COVID-19 prognosis and severity. Therefore, these variables were included in our multivariable Cox proportional regression model. Univariable and multivariable Cox proportional analyses adjusted for age, sex, comorbidities, and smoking and drinking status and the treatments in hospital identified LDL-C dyslipidemia before admission as a risk factor for recurrence of COVID-19 after discharge in the Chengdu cohort (RR: 2.708, 95% CI: 1.283, 5.718; and RR: 2.51, 95% CI: 1.19, 5.29, respectively) (*p* < 0.05, **Table 3**). Unfortunately, Cox proportional analyses showed that the lipid-lowering therapy had no significant effect on the recurrence after discharge (RR: 1.609, 95% CI: 0.771, 3.355, *p=* 0.205).

**Table 3:** Risk factors before admission associated with the primary endpoints including the recurrence after discharge or in-hospital death.

	Univariable RR (95% CI)	p value	Multivariable RR (95% CI)	p value
**Chengdu cohort**				
TC dyslipidemia (*vs.* not)	1.335 (1.014,2.495)	0.008		
TG dyslipidemia (*vs.* not)	0.856 (0.468,1.568)	0.615		
HDL-c dyslipidemia (*vs.* not)	0.037 (0.000,4.883)	0.186		
LDL-c dyslipidemia (*vs.* not)	2.708 (1.283,5.718)	0.009	2.51 (1.19,5.29)	0.006
Lipid-lowering therapy (*vs.* not)	1.609 (0.771,3.355)	0.205		
**Wuhan cohort**				
TC dyslipidemia (*vs.* not)	1.655 (0.746,3.674)	0.216		
TG dyslipidemia (*vs.* not)	2.428 (1.407,4.189)	0.001	1.92 (1.08,3.60)	0.016
HDL-c dyslipidemia (*vs.* not)	1.535 (0.841,2.802)	0.163		
LDL-c dyslipidemia (*vs.* not)	1.403 (0.629,3.131)	0.408		
Lipid-lowering therapy (*vs.* not)	0.849 (0.460,1.564)	0.599		

The data are presented as the risk ratio (RR) (95% CI). We performed Cox proportional hazard regression to determine the potential risk factors associated with the endpoint during time from illness onset to follow-up.

The RRs for endpoints were adjusted for age (continuous variables), BMI, males (*vs.* female), smoking status (*vs.* not present), and drinking status (*vs.* not present) and various kinds of comorbidities (e.g., chronic renal disease, chronic pulmonary disease, hypertension, heart disease, hepatic disease), and laboratory findings (e.g., lymphopenia, and elevated white blood cell count, ALT, AST, high lactate dehydrogenase, high-sensitivity cardiac troponin I, d-dimer, C-reactive protein, creatinine, and procalcitonin) and treatments in hospital.

ALT, alanine aminotransferase; AST, aspartate aminotransferase; TC, total cholesterol; TG, triglycerides; HDL-C, high-density lipoprotein cholesterol; LDL-C, low-density lipoprotein cholesterol.

In the Wuhan cohort, the survival rate of patients with TG dyslipidemia with or without lipid-lowering therapy was lower compared to that of patients without any dyslipidemia, based on a log-rank test (X^2 =^ 6.907, *p* = 0.0361) (**Figure 2B**). Univariable Cox proportional analysis showed that the risk of in-hospital death was found to be higher in patients with TG dyslipidemia before admission. After adjusting for basic variables and the treatments, TG dyslipidemia was risk factor for in-hospital death (RR: 1.92, 95% CI: 1.08, 3.60, *p* = 0.016) (**Table 3**).

We also investigated the associations of components of dyslipidemia before admission with secondary clinical endpoints, including rates of ICU admission and invasive ventilation (**Table 4**). Univariable and multivariable logistic regression models were used to determine risk factors. In the Chengdu cohort, univariable logistic regression showed that there was no component of dyslipidemia associated with the requirement for mechanical ventilation. However, TC or LDL-C dyslipidemia or use of lipid-lowering therapy [odds ratio (OR): 3.574, 95% CI: 1.452, 8.797, *p* = 0.006; 9.849, 95% CI: 2.086, 46.503, *p* = 0.004; 3.364, 95% CI: 1.080, 10.481, *p* = 0.036; respectively] was associated with a higher rate of admission to the ICU. After adjusting, only LDL-C dyslipidemia was significantly associated with ICU admission in multivariable logistic regression (OR: 10.073, 95% CI: 2.073, 48.948, *p* = 0.004). In the Wuhan cohort, univariable logistic regression showed that only TC dyslipidemia was associated with risk of ICU admission (OR: 2.569, 95% CI: 1.218, 8.072, *p* = 0.016). In a multivariable logistic regression model, only TC dyslipidemia was independently associated with ICU admission (OR: 8.147, 95% CI: 1.662, 39.927, *p* = 0.010) (**Table 3**). Overall, the univariate and multivariable models showed that, in both cohorts, patients with dyslipidemia on admission were more likely to experience worse progression and recurrence of COVID-19.

**Table 4:** Risk factors before admission associated with the secondary endpoint after admission.

	Mechanical ventilation				Intensive care unit admission			
	Univariable OR (95% CI)	p value	Multivariable OR (95% CI)	p value	Univariable OR (95% CI)	p value	Multivariable OR (95% CI)	p value
**Chengdu cohort**								
TC dyslipidemia (*vs.* not)	0.436 (0.053, 3.6)	0.441			3.574 (1.452,8.797)	0.006		
TG dyslipidemia (*vs.* not)	0.518 (0.105, 2.551)	0.419			1.312 (0.630,2.732)	0.468		
HDL-c dyslipidemia (*vs.* not)	0.665 (0.101, 2.345)	0.674			0.195 (0.042,0.904)	0.057		
LDL-c dyslipidemia (*vs.* not)	0.458 (0.089, 1.333)	0.788			9.849 (2.086,46.503)	0.004	10.073 (2.073,48.948)	0.004
Lipid-lowering therapy (*vs.* not)	0.865 (0.102, 7.35)	0.894			3.364 (1.080,10.481)	0.036		
**Wuhan cohort**								
TC dyslipidemia (*vs.* not)	1.970 (0.665, 5.842)	0.221			2.569 (1.218,8.072)	0.016	8.147 (1.662,39.927)	0.010
TG dyslipidemia (*vs.* not)	1.427 (0.671, 3.035)	0.356			2.090 (0.922,4.735)	0.077		
HDL-c dyslipidemia (*vs.* not)	1.266 (0.651, 2.461)	0.487			1.060 (0.495,2.269)	0.881		
LDL-c dyslipidemia (*vs.* not)	1.970 (0.665, 5.842)	0.221			2.569 (0.818,8.072)	0.106		
Lipid-lowering therapy (*vs.* not)	0.959 (0.476, 1.933)	0.907			1.140 (0.515,2.524)	0.747		

The data are presented as the odds ratio (OR) (95% CI). We performed logistic regression model to determine the potential risk factors associated with the composite clinical outcomes in hospital.

The ORs for endpoints were adjusted for age (continuous variables), BMI, males (*vs.* female), smoking status (*vs.* not present), and drinking status (*vs.* not present) and various kinds of comorbidities (e.g., chronic renal disease, pulmonary disease, hypertension, heart disease, hepatic disease), and laboratory findings (e.g., lymphopenia, and elevated white blood cell count, ALT, AST, high lactate dehydrogenase, high-sensitivity cardiac troponin I, d-dimer, C-reactive protein, creatinine, and procalcitonin) and treatments in hospital.

ALT, alanine aminotransferase; AST, aspartate aminotransferase; TC, total cholesterol; TG, triglycerides; HDL-C, high-density lipoprotein cholesterol; LDL-C, low-density lipoprotein cholesterol.

### The Lipid Level Change During the COVID-19 Courses

**Figure 3:**
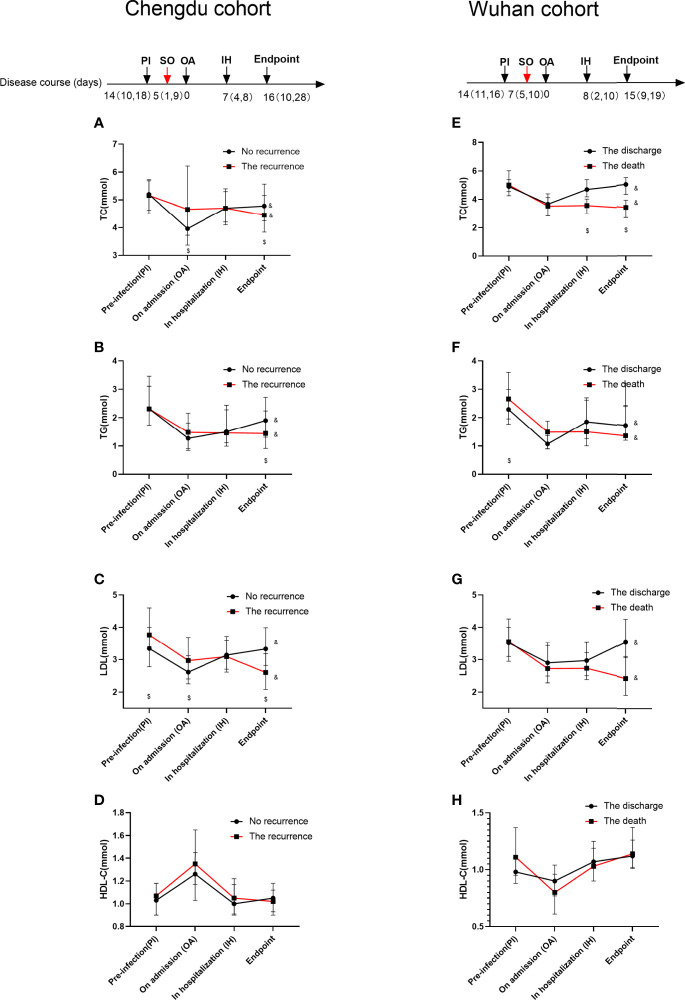
Lipid profiles during the disease course for COVID-19 patients in the Chengdu and Wuhan cohorts. The data are presented as median (IQR). The paired non-parametric test is used to compare the difference among different stages in the same cohort subgroup of patients. The Mann–Whitney U test is used to compare the difference between the two groups. $p < 0.05 as compared with the levels between the discharge group and the recurrence or death of patients group in the same stage. &p < 0.05 as compared with the levels in the four stages of the same cohort. The TC, TG, LDL-C, and HDL-C changes during the disease course in the Chengdu cohort are shown in **(A–D)**. The lipid profile changes during the disease course in the Wuhan cohort are shown in **(E–H)**. The red arrow indicates the time of symptom onset (SO); the black arrows indicate the four stage times including pre-infection (PI), on admission (OA), in hospitalization (IH), and the time of discharge or in-hospital death (endpoint).

We next analyzed the serum lipid levels (in mmol/L) of the patients in four stages during their entire courses of the disease: prior to infection by SARS-CoV-2 as the before admission, on admission, in hospitalization, and the time of discharge or in-hospital death. The average timeline of disease course was calculated and is shown in **Figure 3**. We found that the poor progression of COVID-19 (the in-hospital death or recurrence) patients was more likely to have higher lipid levels in TC, TG, and LDL-C prior to infection and on admission (**Figures 3A, C, F** **)** as compared with those that survived or with no recurrence after discharge patients. For example, the LDL-C level was higher in the recurrence after discharge patients compared with the no recurrence after discharge patients prior to infection [the recurrence: 3.77 (3.41,4.59); no recurrence: 3.34 (2.78,4.02)]. These lipid levels declined in the hospitalization and the time of discharge or in-hospital death as compared to the levels prior to infection, and a larger reduction was shown in the last stage for the poor progression of COVID-19 (the in-hospital death or recurrence after discharge) patients; however, the lipid level in the discharge patients without recurrence and the survived patients trended to return to the level prior to infection (**Figures 3A–C, E–G**). Unlike other lipid levels, HDL-C levels remained relatively steadily during the entire courses of the disease. We found that there were no relationships between dyslipidemia on admission and COVID-19 severity in either cohort (**Supplementary Tables S2, S3**). However, the lipid levels in the hospitalization and the time of discharge or death such as TC and LDL-C were negatively associated with the severity of disease in the Wuhan cohort. The detailed information is provided in **Supplementary Table S4**. We also compared the differences in lipid levels for multiple monitoring during the disease course between both cohorts of patients based on disease severity, as shown in **Supplementary Table S5**. We found that there were significant differences in lipid levels between both cohorts, but differences were stage-special and related to disease severity. For example, many lipid levels in the Chengdu cohort on admission in each disease severity were higher than those in the Wuhan cohort.

## Discussion

This retrospective two-center cohort study revealed, after adjusting for demographics, self-reported comorbidities and laboratory results on admission; high lipid level before admission was a major risk factor for ICU admission, in-hospital death, and recurrence of COVID-19 after discharge (as confirmed by detection of viral RNA in oropharyngeal swab samples), while the lipid level may be lower along with the COVID-19 course during the latter stages compared to that on admission in both cohorts and became lower in COVID-19 patients with the poor clinical endpoints. Moreover, we found that the lipid levels were associated with the inflammation and hepatic and renal organ function in COVID-19 patients, which indicates that COVID-19 may have substantial impact on the lipid metabolism change.

**Figure 4:**
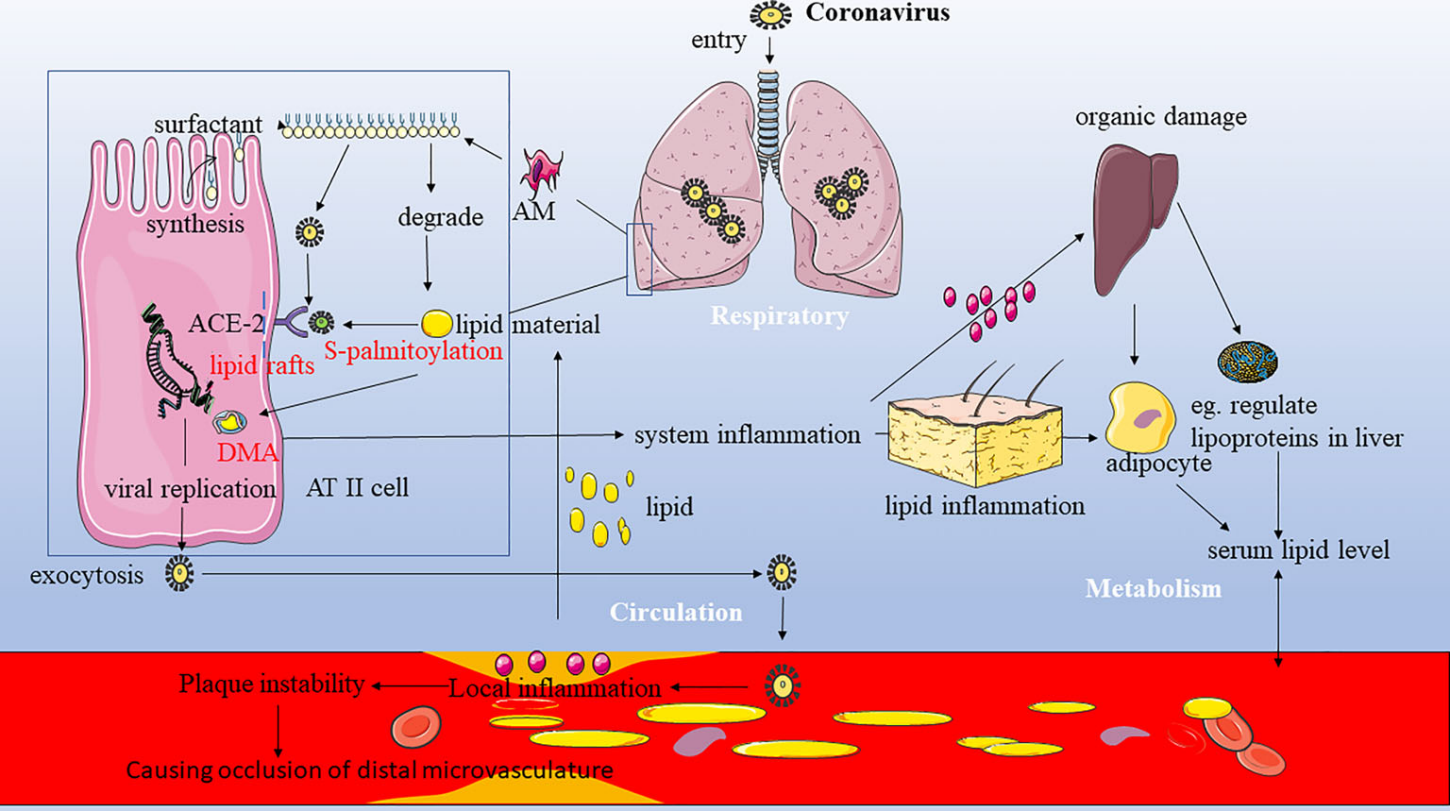
The potential bidirectional interaction between the host lipids and COVID-19 and the role of lipid in viral entry and invasion. Combining our results above with the viewpoints from previous studies (Guan et al., 2020; Mehra et al., 2020; Radenkovic et al., 2020; Alketbi et al., 2021), we have provided the model figure to help us understand the underlying mechanisms to explain the bidirectional interaction between host lipid metabolism and SARS-CoV-2 which led to the poor progression of COVID-19. ACE-2, angiotensin-converting enzyme 2; AM, alveolar macrophage; ATII, alveolar type II cells; DMV, double-membrane vesicle.

Combining our results above with the viewpoints from previous studies (Guan et al., 2020; Mehra et al., 2020; Radenkovic et al., 2020; Alketbi et al., 2021), we have provided the model figure (**Figure 4**) to help us understand the underlying mechanisms to explain the bidirectional interaction between the host lipid metabolism and SARS-CoV-2 which led to the poor progression of COVID-19. Our findings are in accordance with previous reports on the impact of cardiovascular metabolic comorbidities on COVID-19 outcomes (Guan et al., 2020). Our multivariable analysis suggested that, in particular, dyslipidemia before admission predisposes COVID-19 patients to mortality in hospital. Dyslipidemia has been reported to exacerbate a significantly higher risk to atherothrombotic or other cardiovascular complications (Durrington, 1998). Thus, the patients with dyslipidemia were more prone to COVID-19 with worse prognosis. Previous studies have established acute respiratory viral infections such as those caused by influenza virus and coronaviruses to trigger cardiovascular complications such as acute coronary syndrome, myocarditis, arrhythmias, and heart failure acceleration (Nguyen et al., 2016). Several mechanisms have been suggested for the increase in cardiac injury seen in COVID-19 patients that the cytokine storm caused by COVID-19 may result in the development of fulminant myocarditis (Madjid et al., 2020). Shi et al. further proposed that the acute inflammatory response that has been reported in COVID-19 might exacerbate the inflammatory activity within atherosclerotic plaques as well as cause endothelial dysfunction, finally resulting in atherothrombotic complications, as shown in **Figure 4** (Shi et al., 2020).

In addition, dyslipidemia was a significant risk factor for the recurrence of COVID-19 after discharge in our study, although to our knowledge, there are very few longitudinal studies to predict the value of the baseline lipid profile for the poor progression of COVID-19. One possible reason was that the result of the SARS-CoV-2 RNA test in COVID-19 probably depends on the viral load of the specimen. Moreover, the lipids play crucial roles as a source of energy and in signaling in the SARS-CoV-2 life cycle, particularly in viral entry, invasion, and replication (Alketbi et al., 2021). Alveolar type II (ATII) cells have long been known to bind and take up many kinds of lipids including HDL, LDL, and very LDL (VLDL) and to re-secrete phospholipids and cholesterol, which are mostly used up to support the viral infection and utilize the host lipids for their own propagation at the alveolar cells (Alketbi et al., 2021). Several mechanisms have been proposed to explain this propagation in ATII cells. Firstly, surfactant is synthesized within the ATII epithelial cells, which is actively being secreted, and its materials are constantly being exchanged and recycled into the ATII cells to maintain the constant surfactant pool size, while some materials can be degraded by alveolar macrophages (AM) to lipids that can support the viral infection (Goerke, 1998). One recent finding by Cao et al. showed that Spike protein-mediated viral infection was mainly found in SPC+ and LDLR+ pneumocytes and macrophages in the lungs of an animal model, further supporting the statement (Cao et al., 2021). Secondly, viral attachment to the host cell receptor is lipid-dependent. The entry of SARS-CoV-2 is mediated by the binding of the viral spike (S) protein to the ACE2 receptor, which is localized in cholesterol-rich microdomains within the lipid rafts (Lu et al., 2008). On the other hand, S-palmitoylation is a unique protein lipidation process essential for viral invasion. S-palmitoylation of SARS-CoV-2 S protein has been reported to facilitate its anchor and fusion with the host cellular membrane receptor (Santos-Beneit et al., 2020). Thirdly, coronaviruses hijack the host cells for the formation of the replication and transcription complex, the double membrane vesicles (DMVs). DMV formation requires specific lipid compositions. Formation of DMVs is a key factor for viral replication which provides a favorable barrier to protect the viral replication compartments from host innate immune responses (Strating and van Kuppeveld, 2017).On the other hand, in our study, there were some potential impacts of COVID-19 on host lipid metabolism. We attempted to explain this result. Furthermore, in our multivariate analysis of the Chengdu cohort, inflammatory biomarkers (e.g., CRP and procalcitonin) and biomarkers of impaired hepatic and renal function (elevated AST, ALT, and creatinine) on admission were negatively and positively associated with lipid levels, respectively (**Table 2**). The changes in HDL-c levels during the disease course showed different patterns in the Chengdu cohort as compared to the Wuhan cohort. However, there was no significant difference between two different clinical endpoints in each cohort (**Figure 3**). Thus, unlike the other three lipids, the HDL-C levels may not be associated with the COVID-19 endpoint during its disease course. We are well aware of many factors such as sex, age, different diet habits, and other confounding factors; even the disease severity and different treatments in two samples from the two cohorts could affect the level of lipid profiles, which indicated that COVID-19 had a relatively moderate impact on the HDL-C level. The detailed possible reasons for this discrepancy found in our work were unclear, which need more study to explore the relationship and its underlying mechanism between the HDL-C level and COVID-19.

Inflammation and impaired organ function are two critical aspects of COVID-19 infection (Tay et al., 2020). With greater severity of COVID-19, biomarkers of inflammation, and of impaired hepatic and renal function, were also often increased (**Supplementary Tables S2, S3**). Accordingly, the previous studies showed that other virus infections could influence the host lipid metabolism and change the serum lipid level. For example, hepatitis B patients in the cirrhosis phase have lower levels of HDL-C and LDL-C (Cao et al., 2019). It has also been reported that proinflammatory cytokines such as TNF-a and IL-6 could modulate lipid metabolism by altering liver function and thereby partly reducing lipid biosynthesis in HIV infection patients (Funderburg and Mehta, 2016). In addition, the progression in SARS-CoV2 infection may be associated with the leaking of plasma lipids and cholesterol into the alveolar space through altering vascular permeability, causing a leakage of cholesterol molecules into tissues, such as alveolar spaces, to form exudate (Kuiken et al., 2003).

The previous review studies seemingly indicated the important role of statin therapy in preventing cellular entry of coronavirus, which can guide new therapies that may be directed to SARS-CoV-2 (Katsiki et al., 2020). The reasons for the apparent failure in our work to demonstrate a benefit are unclear but include the potential sources of error inherent in all observational methodologies. One important reason was that the lack of specific indications for the prescription and the dose may influence our results. Another fact was that SARS-CoV might also use different receptors to enter the host cell and the possible effects of statin, mainly *via* inhibition of special receptors such as LDL-receptors (LDL-R) (Khademi et al., 2018), resulting in the absence of any beneficial biological effect regarding statin therapy on the COVID-19. Furthermore, it has been suggested that statin acts differently in ARDS patients at different statuses (hyper-inflammatory *vs.* hypo-inflammatory) (Bos et al., 2017). Further investigation is needed on the role of use of lipid-lowering therapy among patients with COVID-19 infections.

Patients discharged after testing negative for COVID-19 may still be at risk of recurrence and thus could still be capable of spreading the virus (Hoang et al., 2020). Some patients tested turned positive again when assessed by an oropharyngeal swab test for SARS-CoV-2 RNA. This could be due to a previous false-negative test result, which may occur due to sampling error, physician inexperience, or a low quantity of virus (Xiao et al., 2020). Performing multiple tests on the same patient could be an effective approach to achieve more reliable SARS-CoV-2 RNA results (Pujadas et al., 2020). To reduce the risk of COVID-19 transmission, recovering patients should be tested regularly, particularly if they have risk factors for relapse such as baseline dyslipidemia.

This cohort study, however, had some limitations. First, although we adjusted for several common confounders, factors such as exercise and dietary habits were not taken into consideration. Second, data on recurrence of COVID-19 after discharge in the Wuhan cohort were not available, due to the heavy burden on the healthcare system during the early period of the outbreak. In contrast, Chengdu was not considered an endemic area so more medical resources were available to treat and follow up COVID-19 patients after discharge. Thirdly, the different type and dose of lipid-lowering therapy prescription may confound the relationship between treatment and endpoints; thus, additional large-scale, well-designed randomized controlled trials are required to identify the effect of lipid-lowering therapy on COVID-19 infection.

## Conclusions

This study provided robust evidence that baseline dyslipidemia is a significant risk factor for ICU admissions, and with mortality and the risk of oropharyngeal swab tests for SARS-CoV-2 RNA returning positive after discharge. Importantly, our findings also allude to a pathophysiological lipid profile in COVID-19, which enhance our understanding of the mechanisms underlying dyslipidemia.

## Data Availability Statement

The original contributions presented in the study are included in the article/**Supplementary Material**. Further inquiries can be directed to the corresponding authors.

## Ethics Statement

The studies involving human participants were reviewed and approved by the Sichuan Provincial People’s Hospital ethics committee. The patients/participants provided their written informed consent to participate in this study.

## Author Contributions

XZ, BinL, GH, and JF had full access to all data in the study and took responsibility for the integrity of the data and the accuracy of the data. Study design: XZ. Data collection: HJ, JH, TH, and BinL. Statistical analysis: XZ. Manuscript draft: ZM and BilL. All authors contributed to the article and approved the submitted version.

## Funding

This research received the Youth Fund of Sichuan Medical Association (Q19018); the Open Research Project of Shanghai Key Laboratory of Sleep Disordered Breathing (SHKSDB-KF-20-03); Youth Fund of Sichuan Provincial People’s Hospital (2021QN03); and Sichuan Provincial Bureau of Cadre Health Care Project (2021-210); National Natural Science Foundation of China (82101206).

## Conflict of Interest

The authors declare that the research was conducted in the absence of any commercial or financial relationships that could be construed as a potential conflict of interest.

## Publisher’s Note

All claims expressed in this article are solely those of the authors and do not necessarily represent those of their affiliated organizations, or those of the publisher, the editors and the reviewers. Any product that may be evaluated in this article, or claim that may be made by its manufacturer, is not guaranteed or endorsed by the publisher.

## Abbreviations

COVID-19, coronavirus disease 2019; SARS-CoV-2, severe acute respiratory syndrome corona virus 2; COPD, chronic obstructive pulmonary disease; AST, aspartate aminotransferase; ALT, alanine aminotransferase; CRP, C-reactive protein; BMI, body mass index; ICU, intensive care unit; TC, total cholesterol; TG, triglycerides; HDL-C, high-density lipoprotein cholesterol; LDL-C, low-density lipoprotein cholesterol.
