# Supplementary material for: Altered Lipid Profile Is a Risk Factor for the Poor Progression of COVID-19: From Two Retrospective Cohorts
Source: Front Cell Infect Microbiol. 2021 Sep 30;11:712530. doi: 10.3389/fcimb.2021.712530 (PMC8515140; doi:10.3389/fcimb.2021.712530)
Supplement: Supplementary file 1 [file Table_1.doc]

Table S1. The lipid profiles and comorbidities in COVID-19 patients.

|  | Without comorbidities | With comorbidities | | P |
| --- | --- | --- | --- | --- |
|  | Without obesity | With obesity |  | |
| TC | 3.99±1.10 | 3.87±0.99 | 0.349 | |
| TG | 1.41±0.81 | 1.53±0.87 | 0.232 | |
| HDL-c | 1.09±0.35 | 1.00±0.30 | 0.032 | |
| LDL-c | 2.90±0.74 | 2.93±0.69 | 0.724 | |
|  | Without hypertension | With hypertension |  | |
| TC | 3.94±1.04 | 4.06±1.23 | 0.374 | |
| TG | 1.33±0.77 | 1.64±0.95 | 0.008 | |
| HDL-c | 1.07±0.32 | 1.05±0.40 | 0.725 | |
| LDL-c | 2.91±0.72 | 2.92±0.80 | 0.969 | |
|  | Without heart disease | With heart disease |  | |
| TC | 4.00±1.11 | 3.58±0.76 | 0.116 | |
| TG | 1.45±0.84 | 1.32±0.75 | 0.538 | |
| HDL-c | 1.07±0.34 | 0.96±0.35 | 0.178 | |
| LDL-c | 2.93±0.75 | 2.70±0.52 | 0.212 | |
|  | Without diabetes | With diabetes |  | |
| TC | 3.93±1.07 | 4.43±1.28 | 0.019 | |
| TG | 1.40±0.83 | 1.81±0.79 | 0.011 | |
| HDL-c | 1.07±0.34 | 1.04±0.37 | 0.701 | |
| LDL-c | 2.88±0.71 | 3.27±0.93 | 0.007 | |
|  | Without renal disease | With renal disease |  | |
| TC | 3.96±1.10 | 3.42±0.49 | 0.218 | |
| TG | 1.43±0.84 | 1.73±0.39 | 0.397 | |
| HDL-c | 1.07±0.34 | 0.84±0.36 | 0.104 | |
| LDL-c | 2.92±0.74 | 2.58±0.53 | 0.366 | |
|  | Without pulmonary disease | With pulmonary disease |  | |
| TC | 3.97±1.09 | 3.90±1.21 | 0.818 | |
| TG | 1.44±0.83 | 1.38±0.80 | 0.819 | |
| HDL-c | 1.06±0.34 | 1.20±0.32 | 0.191 | |
| LDL-c | 2.93±0.74 | 2.54±0.70 | 0.088 | |
|  | Without hepatic disease | With hepatic disease |  | |
| TC | 3.97±1.11 | 4.00±0.91 | 0.907 | |
| TG | 1.44±0.83 | 1.46±0.77 | 0.910 | |
| HDL-c | 1.06±0.34 | 1.18±0.34 | 0.182 | |
| LDL-c | 2.92±0.75 | 2.71±0.50 | 0.257 | |
|  | Without intracranial disease | With intracranial disease |  | |
| TC | 3.96±1.09 | 4.94±1.04 | 0.048 | |
| TG | 1.44±0.84 | 1.54±0.25 | 0.782 | |
| HDL-c | 1.06±0.34 | 1.18±0.57 | 0.450 | |
| LDL-c | 2.90±0.73 | 3.68±0.56 | 0.020 | |

Continuous data are presented as the means ± standard deviation (SD).

Differences among the subgroups were examined using the Mann-Whitney U test or t test according to the characteristics of the data distribution.

Abbreviations: TC, total cholesterol; TG, triglycerides; HDL-C, high-density lipoprotein cholesterol; LDL-C, low-density lipoprotein cholesterol.
