# Supplementary material for: Altered Lipid Profile Is a Risk Factor for the Poor Progression of COVID-19: From Two Retrospective Cohorts
Source: Front Cell Infect Microbiol. 2021 Sep 30;11:712530. doi: 10.3389/fcimb.2021.712530 (PMC8515140; doi:10.3389/fcimb.2021.712530)
Supplement: Supplementary file 2 [file Table_2.doc]

Table S2. The lipid profiles and laboratory findings on admission according to COVID-19 severity in Chengdu cohort.

|  | Mild cases | Moderate cases | Severe cases | Critical cases |  |
| --- | --- | --- | --- | --- | --- |
|  | n=19 | n=85 | n=16 | n=13 | P for linear trend |
| TC dyslipidemia, % | 21.1 | 21.2 | 12.5 | 15.4 | 0.491 |
| TG dyslipidemia, % | 18.2 | 35.3 | 31.2 | 30.8 | 0.539 |
| HDL-c dyslipidemia, % | 10.5 | 7.1 | 37.5 | 7.7 | 0.239 |
| LDL-c dyslipidemia, % | 5.3 | 12.9 | 6.2 | 0 | 0.408 |
| White blood cell count, 10⁹/L, % |  |  |  |  | 0.001 |
| <4 | 9.1 | 8.2 | 12.5 | 0 |
| >10 | 4.5 | 5.9 | 6.2 | 16.2 |
| Lymphocyte count, 10⁹/L, % |  |  |  |  | 0.001 |
| <0·8 | 0 | 15.3 | 56.2 | 38.5 |
| >4 | 4.5 | 0 | 0 | 18.5 |
| Platelet count, 10⁹ /L, % |  |  |  |  | 0.361 |
| <100 | 4.5 | 2.4 | 12.5 | 7.7 |
| >300 | 4.5 | 9.4 | 0 | 15.4 |
| C-reactive protein, >5mg/L, % | 31.8 | 37.6 | 87.5 | 76.9 | 0.001 |
| D-dimer, >0.5mg/L, % | 4.8 | 7.1 | 25 | 69.2 | 0.001 |
| Lactate dehydrogenase, U/L, % |  |  |  |  | 0.001 |
| <109 | 9.1 | 0 | 0 | 0 |
| >109 | 0 | 9.4 | 56.2 | 69.2 |
| High-sensitive cardiac troponin I, >0.04ng/mL, % | 0.02 | 0.21 | 0.11 | 0.12 | 0.356 |
| Procalcitonin, >0.5μg/L, % | 0 | 0 | 6.2 | 15.4 | 0.001 |
| Alanine aminotransferase, >40U/L, % | 13.6 | 27.1 | 31.2 | 30.8 | 0.026 |
| Aspartate aminotransferase, >40U/L, % | 9.1 | 9.4 | 37.5 | 15.4 | 0.092 |
| Creatinine, μmol/L, % |  |  |  |  | 0.001 |
| <40 | 4.5 | 3.5 | 6.2 | 0 |
| >133 | 0 | 0 | 0 | 23.1 |

The data are presented as the as percentages.

The linear-by-linear association test was used for dichotomous variables.

Abbreviations: TC, total cholesterol; TG, triglycerides; HDL-C, high-density lipoprotein cholesterol; LDL-C, low-density lipoprotein cholesterol.
