# Supplementary material for: Altered Lipid Profile Is a Risk Factor for the Poor Progression of COVID-19: From Two Retrospective Cohorts
Source: Front Cell Infect Microbiol. 2021 Sep 30;11:712530. doi: 10.3389/fcimb.2021.712530 (PMC8515140; doi:10.3389/fcimb.2021.712530)
Supplement: Supplementary file 3 [file Table_3.doc]

Table S3. The lipid profiles and laboratory findings on admission according to COVID-19 severity in Wuhan cohort.

|  | Mild cases | Moderate cases | Severe cases | Critical cases |  |
| --- | --- | --- | --- | --- | --- |
|  | n=0 | n=117 | n=21 | n=55 | P for linear trend |
| TC dyslipidemia, % | NS | 6.8 | 4.8 | 10.9 | 0.395 |
| TG dyslipidemia, % | NS | 12 | 36.4 | 19.3 | 0.053 |
| HDL-c dyslipidemia, % | NS | 58.1 | 61.9 | 65.5 | 0.355 |
| LDL-c dyslipidemia, % | NS | 6.8 | 4.8 | 10.9 | 0.395 |
| White blood cell count, 10⁹/L, % |  |  |  |  | 0.004 |
| <4 | NS | 28.2 | 18.2 | 20.7 |
| >10 | NS | 1.7 | 9.1 | 20.7 |
| Lymphocyte count, 10⁹/L, % |  |  |  |  | 0.002 |
| <0·8 | NS | 60.7 | 77.3 | 82.8 |
| >4 | NS | 9.3 | 2.7 | 7.2 |
| Platelet count, 10⁹ /L, % |  |  |  |  | 0.052 |
| <100 | NS | 18.8 | 27.3 | 29.3 |
| >300 | NS | 2.6 | 9.1 | 5.2 |
| C-reactive protein, >5mg/L, % | NS | 80.3 | 90.9 | 96.6 | 0.003 |
| D-dimer, >0.5mg/L, % | NS | 43.8 | 71.4 | 82.5 | 0.001 |
| Lactate dehydrogenase, U/L, % |  |  |  |  | 0.001 |
| <109 | NS | 1.7 | 0 | 0 |
| >109 | NS | 39.3 | 63.6 | 82.8 |
| High-sensitive cardiac troponin I, >0.04ng/mL, % | NS | 4 | 16.7 | 37.5 | 0.001 |
| Procalcitonin, >0.5μg/L, % | NS | 20 | 59 | 75.9 | 0.001 |
| Alanine aminotransferase, >40U/L, % | NS | 16.2 | 13.6 | 27.6 | 0.091 |
| Aspartate aminotransferase, >40U/L, % | NS | 15.4 | 31.8 | 46.6 | 0.001 |
| Creatinine, μmol/L, % |  |  |  |  | 0.001 |
| <40 | NS | 0.9 | 0 | 6.9 |
| >133 | NS | 1.7 | 13.6 | 36.2 |

The data are presented as percentage.

The linear-by-linear association test was used for dichotomous variables.

Abbreviations: TC, total cholesterol; TG, triglycerides; HDL-C, high-density lipoprotein cholesterol; LDL-C, low-density lipoprotein cholesterol.
