# Supplementary material for: Altered Lipid Profile Is a Risk Factor for the Poor Progression of COVID-19: From Two Retrospective Cohorts
Source: Front Cell Infect Microbiol. 2021 Sep 30;11:712530. doi: 10.3389/fcimb.2021.712530 (PMC8515140; doi:10.3389/fcimb.2021.712530)
Supplement: Supplementary file 4 [file Table_4.docx]

Table S4. Lipid profiles during disease course for COVID-19 patients in Chengdu and Wuhan cohorts.

|  | Chengdu cohort | | | | | | | |
| --- | --- | --- | --- | --- | --- | --- | --- | --- |
|  | The discharge (n=90) | | | | The recurrence (n=43) | | | |
|  | Pre-infection | admission | hospitalization | discharge | Pre-infection | admission | hospitalization | recurrence |
| Disease course (days) | 14(11,18) | 5(2,10) | 9(15,26) |  | 14(10,19) | 4(0,9) | 19(12,32) |  |
| TC mmol/L | 5.20(4.50,5.69) | 3.96(3.37,4.71)$ | 4.7(4.1,5.3) | 4.78(4.24,5.57)$ & | 5.16(4.62,5.73) | 4.66(3.73,6.22)$ | 4.7(4.2,5.4) | 4.45(3.84,5.17)$ & |
| TG | 2.31(1.73,3.11) | 1.27(0.9,1.81) | 1.5(1.11,2.28) | 1.9(1.31,2.72)$& | 2.31(1.3,3.46) | 1.48(0.84,2.16) | 1.46(0.99,2.44) | 1.44(0.91,2.24)$ & |
| LDL | 3.34(2.78,4.02) | 2.61(2.25,3.12) | 3.14(2.61,3.72) | 3.33(2.82,3.99)$ | 3.77(3.41,4.59) | 2.97(2.41,3.69) | 3.09(2.7,3.6) | 2.6(2.08,3.18)$ & |
| HDL | 1.03(0.9,1.18) | 1.26(1.03,1.45) | 1.005(0.90,1.17) | 1.05(0.93,1.18) | 1.07(0.9,1.18) | 1.35(1.17,1.65) | 1.05(0.91,1.22) | 1.02(0.89,1.12) |
|  | Wuhan cohort | | | | | | | |
|  | The discharge (n=138) | | | | The death in hospital (n=55) | | | |
| mmol/L  Disease course (days) | Pre-infection | admission | hospitalization | discharge | Pre-infection | admission | hospitalization | death |
|  | 14(12,16) | 7(6,10) | 17(12,20) |  | 12(10,16) | 7(5,13) | 8(4,18) |  |
| TC | 4.91(4.26,5.41)& | 3.67(3.15,4.13) | 4.70(4.18,5.4) | 4.87(4.31,5.50)$ | 5.02(4.56,6.01)& | 3.53(2.84,4.39) | 3.57(3,4.03) | 3.39(2.71,3.89)$ |
| TG | 2.29(1.77,3.02)& | 1.07(0.89,1.37) | 1.85(1.25,2.62) | 1.71(1.20,2.42)$ | 2.66(1.96,3.6)& | 1.49(0.94,1.88) | 1.5(1,2.71) | 1.36(0.92,2.13)$ |
| LDL-c | 3.52(2.95,4.02)& | 2.91(2.49,3.44) | 2.97(2.5,3.55) | 3.55(3.07,4.25)$ | 3.56(3.1,4.26)& | 2.72(2.28,3.54) | 2.73(2.38,3.21) | 2.43(1.91,3.11)$ |
| HDL | 0.98(0.88,1.10)& | 0.90(0.77,1.04) | 1.07(0.92,1.25) | 1.125(1.01,1.37) | 1.11(0.95,1.37)& | 0.81(0.61,0.96) | 1.03(0.9,1.19) | 1.14(1.02,1.26) |

The data is presented as median (IQR). The paired nonparametric test is used to compare the difference among different stages in the same cohort subgroup patients. Mann-Whitney U test is used to compare the difference between two groups. $ *p*<0.05 as compared with the levels between the discharge group and the recurrence or death patients in the same stage. & p<0.05 as compared with the levels in the four stages of in the same cohort.
