# Supplementary material for: Altered Lipid Profile Is a Risk Factor for the Poor Progression of COVID-19: From Two Retrospective Cohorts
Source: Front Cell Infect Microbiol. 2021 Sep 30;11:712530. doi: 10.3389/fcimb.2021.712530 (PMC8515140; doi:10.3389/fcimb.2021.712530)
Supplement: Supplementary file 5 [file Table_5.docx]

Table S5. The differences in lipid levels among both cohorts of patients for multiple monitoring during disease course based on disease severity.

|  |  | Chengdu cohort | | | | Wuhan cohort | | | |
| --- | --- | --- | --- | --- | --- | --- | --- | --- | --- |
|  |  | Mild (n=19) | Moderate (n=85) | Severe (n=16) | Critical(n=13) | Mild | Moderate (n=117) | Severe (n=21) | Critical (n=55) |
| Pre-infection |  |  |  |  |  |  |  |  |  |
|  | TC | 4.8(4.2,5.63) | 5.21(4.61,5.65) | 5.24(4.38,6.20) | 5.62(4.94,7.19) | NA | 4.96(4.33,5.48) | 4.6(4.12,5.38) | 5(4.52,5.93) |
|  | TG | 1.99(1.2,3.46) | 2.42(1.57,3.45) | 2.055(1.37,3.04) | 1.9(1.6,2.78)$ | NA | 2.26(1.76,3) | 2.56(1.91,3.315) | 2.66(2.04,3.6)$ |
|  | LDL-C | 3.17(2.51,3.84) | 3.68(3.02,4.17) | 3.81(2.88,4.11) | 3.72(2.77,5.27) | NA | 3.58(2.98,4.05) | 3.16(2.86,3.8) | 3.55(3.1,4.22) |
|  | HDL-C | 0.99(0.87,1.15) | 1.07(0.90,1.18) | 0.955(0.9,1.11) | 1.03(0.97,1.24) | NA | 0.99(0.9,1.115) | 0.99(0.89,1.23) | 1.06(0.9,1.33) |
| Admission |  |  |  |  |  |  |  |  |  |
|  | TC | 4.22(3.25,4.94) | 4.29(3.69,5.20)$ | 3.47(3.20,4.95) | 3.67(3.35,4.54) | NA | 3.68(3.13,4.12)$ | 3.34(3.06,4.35) | 3.61(2.99,4.38) |
|  | TG | 0.85(0.7,1.14) | 1.45(0.99,2.18)$ | 1.24(0.9,2.03) | 1.47(1.00,2.01) | NA | 1.07(0.89,1.37)$ | 1.36(0.92,1.83) | 1.3(0.91,1.86) |
|  | LDL-C | 2.88(2.27,3.2) | 2.87(2.33,3.31) | 2.58(2.27,3.11) | 2.44(2.07,2.98) | NA | 2.91(2.49,3.42) | 2.74(2.35,3.56) | 2.81(2.35,3.51) |
|  | HDL-C | 1.33(1.14,1.51) | 1.35(1.14,1.54)$ | 1.045(0.80,1.37)$ | 1.17(1.03,1.42)$ | NA | 0.9(0.79,1.05)$ | 0.81(0.69,0.98)$ | 0.84(0.66,0.97)$ |
| Hospitalization |  |  |  |  |  |  |  |  |  |
|  | TC | 4.7(4,5.3) | 4.8(4.3,5.45) | 4.6(3.875,4.875) | 4.8(4.5,5)$ | NA | 4.74(4.195,5.43) | 4.4(3.975,4.95) | 3.58(3,4.1)$ |
|  | TG | 1.63(1.43,2.9) | 1.42(0.98,2.12)$ | 2.2(1.2775,3.075) | 1.34(1.03,1.61) | NA | 1.82(1.21,2.715)$ | 2.16(1.275,2.78) | 1.61(1,2.53) |
|  | LDL-C | 2.99(2.59,3.53) | 3.16(2.74,3.87) | 2.645(2.415,3.33) | 3.38(2.705,3.73)$ | NA | 3.01(2.515,3.57) | 2.78(2.525,3.415) | 2.66(2.34,3.21)$ |
|  | HDL-C | 0.98(0.87,1.2) | 1.01(0.91,1.21) | 1.025(0.9525,1.15) | 1.05(0.845,1.325) | NA | 1.05(0.93,1.25) | 1.07(0.93,1.285) | 1.03(0.89,1.2) |
| Discharge |  |  |  |  |  |  |  |  |  |
|  | TC | 4.66(4.02,5.87) | 4.66(4.14,5.47) | 4.65(3.62,5.24) | 4.94(4.025,5.615)$ | NA | 4.88(4.335,5.51) | 4.1(3.355,5.23) | 3.55(2.73,4.21)$ |
|  | TG | 1.92(1.23,2.61) | 1.7(1.20,2.53) | 2.14(1.49,2.80) | 1.5(1.165,2.685) | NA | 1.7(1.195,2.34) | 1.76(0.88,2.765) | 1.46(0.94,2.25) |
|  | LDL-C | 3.23(2.01,3.95) | 3.05(2.57,3.84)$ | 2.86(2.58,3.89) | 3.12(2.77,4.13)$ | NA | 3.61(3.095,4.22)$ | 3.11(2.525,3.655) | 2.56(1.96,3.29)$ |
|  | HDL-C | 1.04(0.91,1.31) | 1.05(0.92,1.16)$ | 1.075(0.975,1.2375) | 0.99(0.93,1.085)$ | NA | 1.14(1.02,1.37)$ | 1.06(1.005,1.39) | 1.12(1.01,1.23)$ |

The data is presented as median (IQR). Mann-Whitney U test is used to compare the difference between two groups. $ p<0.05 as compared with the levels between two cohort patients in the same stage. NA, data not available.
